# Supplementary figures and images for: A novel role for Dun1 in the regulation of origin firing upon hyper-acetylation of H3K56
Source: PLoS Genet. 2021 Feb 18;17(2):e1009391. doi: 10.1371/journal.pgen.1009391 (PMC7924802; doi:10.1371/journal.pgen.1009391)

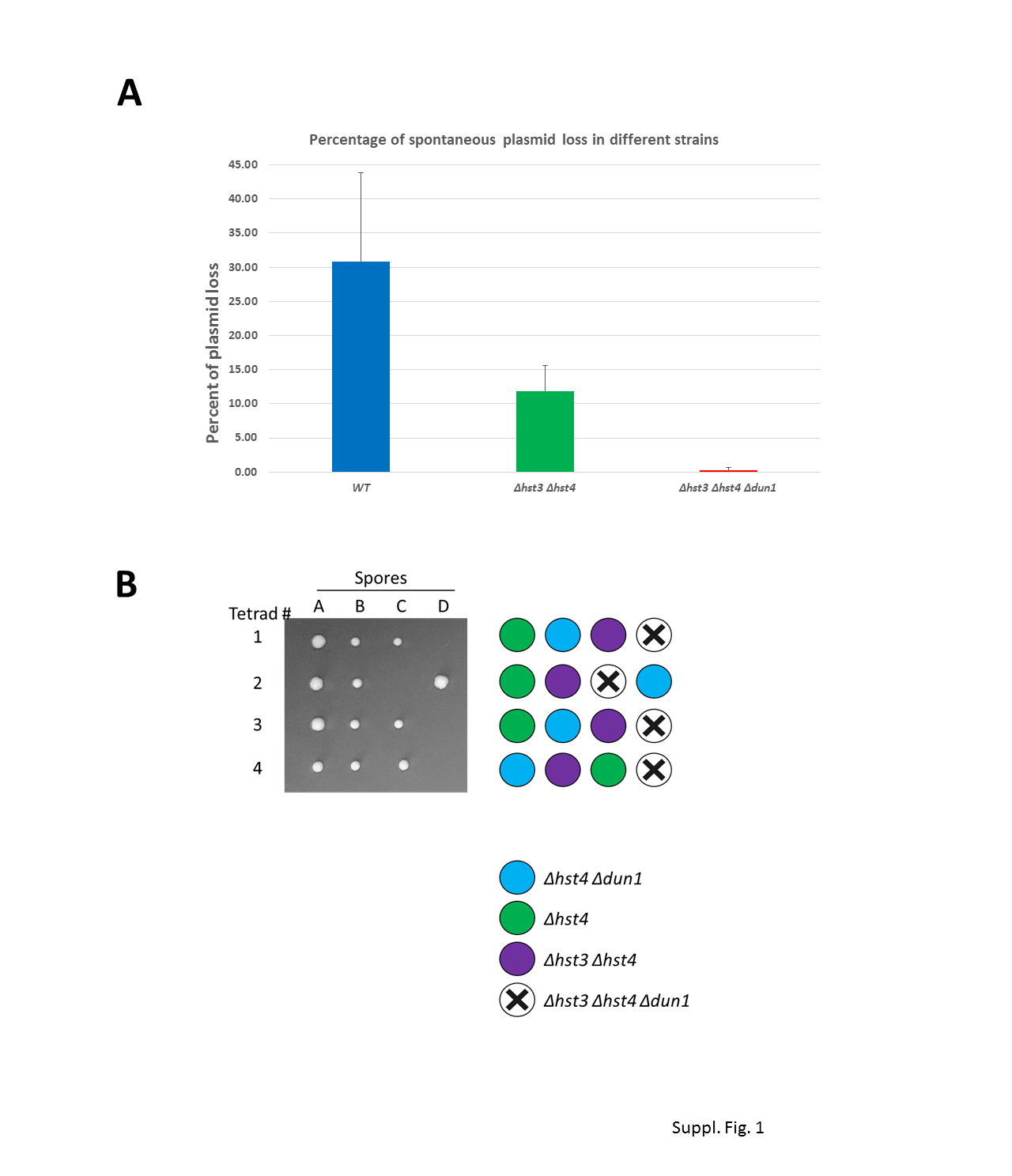

Supplement: S1 Fig — (A) Strains containing Δade2 Δade3 and carrying plasmid pRS316-ADE3-HST3 were grown overnight in selective media, then diluted and plated onto YPD. Following 3 days of growth, YPD plates were replicated onto SD-Ura plates and the colonies were compared for growth between the plates. Most of the colonies that failed to grow on SD–Ura plates were also white, with a negligible percentage of red colonies. (B) Tetrad analysis showing that a triple deletion of HST3, HST4 and DUN1 is inviable. An Δhst3 Δhst4 Δdun1 strain carrying a pURA3-HST3 plasmid was crossed to an Δhst4 strain. Diploids were streaked onto 5-FOA to lose plasmid, and sporulated. Tetrads were dissected under a micromanipulator and their genotype was confirmed by markers. (TIF) [file pgen.1009391.s001.tif]
