# Supplementary material for: A novel role for Dun1 in the regulation of origin firing upon hyper-acetylation of H3K56
Source: PLoS Genet. 2021 Feb 18;17(2):e1009391. doi: 10.1371/journal.pgen.1009391 (PMC7924802; doi:10.1371/journal.pgen.1009391)
Supplement: Attachment — Submitted filename: Point by point1c.docx [file pgen.1009391.s004.docx]

Point-by-point responses (in black) to comments by referees (in blue).

Dear Martin,

Thank you very much for submitting your Research Article entitled 'Novel roles for Dun1 and Mrc1 in the regulation of origin firing upon hyper-acetylation of H3K56' to PLOS Genetics. Your manuscript was fully evaluated at the editorial level and by independent peer reviewers. The reviewers appreciated the attention to an important problem and the thorough genetic analyses done, but requested additional experiments to further support the conclusions as well as textual modifications.  A summary figure presenting the inferred models might also be helpful.  

Dear Sue,

We are submitting a revised version of the manuscript. In response to the referees' comments (see below a point-by-point answer) we have carried out additional experiments to demonstrate our hypothesis, and introduced many changes in the text. The paper is now more focused in the new Dun1 role (for which we present new results demonstrating the model in Figure 7). As we explain, there are very few functions for Dun1 outside its well-characterized role as a regulator of dNTP levels. Hence, we have changed the title accordingly. We have also added a figure with a model (Figure 8). The changes introduced appear in red color in the manuscript.

Reviewer #1: This is beautiful piece of work of the Kupiec lab showing using classical yeast genetics that Dun1 has a so far unknown function controlling Rad53 phosphorylation and in turn late origin firing, as a way to explain the lethality caused by dun1 in hst3 hst4 genetic background that is suppressed by rad53. A similar study is made for mrc1 mutants, but in this case the molecular explanation for the lethality caused by triple mutations seems different and not caused by suppression of late firing. The latter, provides a good example of the complexity of events that can cause lethality of hst3 hst4 mutants and the need to understand the molecular events generated in these double mutants that can compromise viability. It is a manuscript appropriate for PLoS Genetics that researchers in the field will find highly inspiring. I just make a number of suggestions and request few controls that will make the conclusions more convincing by going a step ahead and showing by a minimum molecular analysis the validity of the model proposed.

We thank the reviewer for her/his positive opinion of our manuscript.

Title. Is it really appropriate? I am not sure the manuscript shows that Mrc1regulates origin firing.

We have changed the title to: "A novel role for Dun1 in the regulation of origin firing upon hyper-acetylation of H3K56". This reflects the new, stronger focus on Dun1.

Introduction. It is too long. It could be considerable reduced, in particular page 4 which is more adequate for a high school reader.

Some parts of the Results section can be shortened. Authors write the manuscript reasoning nicely every experiment. However, in a number of cases the premises on which the experiments were based did not fit with the results obtained. This is the case showing that the viability does not depends on the levels of PCNA in chromatin (page 10). There is no need for so many explanations for a negative result.

Similar suggestion on the negative role of Dun1 as a regulator of RNRs’ negative regulators in page 11.

Similar suggestions about the negative result showing no involvement of chromatin remodelers in viability, this part being unnecessarily long (pages 11 & 12)

We have shortened and simplified the explanations in all these passages, and significantly shortened the Introduction, as suggested by the referee.

Westerns in Figs. 1C and 4C do not show the WT. It needs to be shown.

The wt control and all the appropriate mutants are now shown in Figure 1C.

Checkpoint activation of Rad53 after H3K56 hyper-acetylation. ∆rad53 suppresses dun1 lethality but not mrc1. Perhaps ∆mrc1 leads to a lethal intermediate, since Mrc1, in contrast to Dun1 that is a kinase and a regulator, Mrc1 is a structural protein working at forks the absence of which can alter the output of possible RF collapses causing toxic intermediates that would lead to lethality. Do authors consider this to be an option? Discuss.
Hst3 hst4 mutants have been shown to have other phenotypes such as high levels of Rad52 foci and high recombination (Alvaro et al PLoS Genet 2007), synthetic lethality with rad52 (Celic et al, Genetics 2008) and defective sister chromatid recombination repair (Munoz et al, PLoS Genet 2013). It seems that, as likely expected for mutants affecting chromatin, hst3 hst4 creates a pleiotropic vulnerability that makes cells be dependent on functions non-essential in wt cells, like dun1, rad52, mrc1, and more, as summarized in Supp TAble 1. Authors could discuss these other options, in particular for the mrc1 mutation. Wouldn’t make sense to think that lethality caused by Mrc1, which, in contrast to Dun1, plays a physical role at forks and likely at collapsed fork during repair, is related to the accumulation of unrepaired collapsed forks or DSB intermediates? It would help the article to expand Discussion taking into account these facts.

We thank the referee for the suggestions. We have incorporated these ideas into the Discussion (lines 416 and on).

Once authors infer that it is the ability of constitutively fire late origins in ∆rad53 cells what rescues the lethality caused by ∆Dun1, wouldn’t it be necessary to check this? Authors could check that one late origin controlled by Rad53 fires replication at early S phase in dun1 cells via ChIP with a pulse of BrdU using appropriate yeast strains able to incorporate BrdU. Authors should try to add direct evidence to their model on the regulation of origin firing, if they want to be that explicit in title and conclusions.

We have indeed carried out experiments to prove our model (presented in Figure 7B). Quantitative PCR analysis of strains carrying a degron allele of *DUN1* show that inactivation of Dun1 in wild type cells has no effect on an early or on a late-firing origin (usually repressed by Rad53). However, degradation of Dun1 in *Δhst3 Δhst4* cells prevents firing for the late origin, demonstrating that cells devoid of Dun1 indeed are defective in the de-regulation of late origin firing.

While the use of FOA to check the capability of cells losing the DUN1-containing plasmid is perfectly valid, authors could show the tetrad analysis of hst3 hst4 x dun1 and hst3,4 x mrc1, to see that triple mutant spores do not grow. Since mutants of chromatin states usually have pleiotropic phenotypes, affecting also transcription whether or not by direct means, it would be nice to see the phenotype of the hst mutations in FOA-free media.

We have carried out tetrad analysis, and the SL phenotype can be seen on YPD plates in Suppl. Fig. 1B.

It would important to show whether H3K56 mutations mimicking acetylated H3K56 (Q), is dead with dun1, but not mutations mimicking non-acetylated (R or A), to support the conclusions on the specificity of H3K56 acetylation.

We have tested that abolishing the site of acetylation by mutating H3K56 to R suppresses the synthetic lethality between *Δhst3 Δhst4* and *Δdun1*. These results are now presented in Figures 1B and 4B. However, a similar experiment using the H3K56Q allele gave inconsistent and unreliable results, as has been previously also shown for other phenotypes of *Δhst3 Δhst4* cells.

Reviewer #2: In this work, the authors describe synthetic lethal or sick (SS) interactions between either dun1 or mrc1 mutants and hst3∆ hst4∆ mutant. In addition, they uncovered several mutants suppressing hst3∆ hst4∆ mutant growth defect at higher temperatures. Besides known hst3∆ hst4∆ suppressors, such as asf1, they uncovered ctf18∆, rad53∆ and dbf4-3A sld3-38A. These three suppressors can exert their suppressive effects also in cells containing hst3∆ hst4∆ dun1∆. Only ctf18∆ and dbf4-3A sld3-38A has suppressive effect in cells containing hst3∆ hst4∆ mrc1∆.

To understand the SS (dun1∆ or mrc1∆) and suppressive effects (ctf18∆, rad53∆ and dbf4-3A sld3-38A) on hst3∆ hst4∆, the authors performed additional genetic analyses, which ruled out the involvement of cohesion, alteration of PCNA level on chromatin, histone, and dNTP levels.

The authors further used separation of function alleles of dun1 and mrc1, and ctf18 to suggest that Dun1 interaction with Rad53 is not important for the SS with hst3∆ hst4∆, while loss of Ctf18 or Mrc1 binding to Pol epsilon can be relevant.

One strength of the work is the findings of additional hst3∆4∆ genetic interactions. Another strength is the finding that the negative interactions between Dun1∆ and hst3∆4∆ is unrelated to dNTP regulation. The authors suggest that this can mean that Dun1 has a role in origin firing independent of Rad53.

We thank the reviewer for the time taken to go over this work and for appreciating the strengths of our manuscript.

The weakness is the lack of mechanistic insights into any of these interactions - genetic interactions can have more than one interpretation, thus leaving open many possibilities. Following up on one of the many speculations described in the work at a mechanistic level can strengthen the paper. For example, there are many tools available to test whether Dun1 can counter Rad53 in origin firing regulation or if Ctf18 indeed rescues the DNA replication defects seen in hst3∆4∆, hst3∆4∆mrc1∆, or hst3∆4∆dun1∆ cells.

We have re-written the paper, concentrating on the new role found for Dun1. In order to strengthen the mechanistic insights and demonstrate that our model with respect of the function of Dun1 is right, we have carried out additional experiments. Using qPCR and strains carrying a degron allele of Dun1, we have checked the ability of cells experiencing hyper-acetylation of H3K56 and lacking Dun1 to replicate a Rad53-dependent origin on chromosome 14 that is known to fire in hst3∆4∆. We show that indeed Dun1 is essential for the firing, confirming our model (Figure 7B,C).

Also, most genetic tests were done using a plasmid carrying Hst3. For the most critical conclusions, one shall use integrated alleles and degron alleles to verify the findings, as that some of these genetic interactions could be influenced by plasmid instability.

In order to make sure that our assay is not biased due to the use of 5-FOA we have carried out a spontaneous plasmid loss assay that also employed the ability of cells lacking *ADE2* to accumulate a pink pigment. Using this tool we saw that strains lacking Dun1 could not lose their *HST3*-carrying plasmid spontaneously, supporting our conclusions. Furthermore, we show the lethality of strains lacking Dun1 or Mrc1 using tetrad analysis in a manner independent of the *HST3*-carrying plasmid. For the qPCR experiment, we constructed a degron strain of Dun1 and made use of it. These cells are unable to grow on plates containing auxin.

Reviewer #3: This manuscript reports on the genetic relationships between histone acetylation, DNA replication and various aspects of checkpoint signalling in budding yeast. The authors use a robust genetic assay to examine synthetic lethal as well as suppressive relationships in a background of hyperacetylation of new histones. The topic is an interesting one, as the acetylation and subsequent deacetylation of H3 are both required for genome stability, but the reason behind this is not well understood. In this study, the authors describe an essential function of the two checkpoint factors Dun1 and Mrc1 in situations where H3 deacetylation is impeded. They find that the essential function can be suppressed by deletion of another component of the replication fork, Ctf18, and they exclude a series of possible scenarios as reasons, e.g. interactions with the replicative polymerase epsilon or PCNA loading/unloading. Having excluded these factors, they go on an examine a set of checkpoint factors, where they find complicated relations that on one hand differentiate between Dun1 and Mrc1 (i.e. showing non-identical phenotypes) and on the other hand identify a contribution of the checkpoint effector kinase Rad53 as well as an important function of regulating origin firing.
Overall, the experiments shown here appear clean, and the assay they use gives robust data. I have no objections to this experimental approach, as it provides reliable results. I also appreciate the systematic manner in which the authors exclude some potential factors that could be invoked as a model.

We thank the referee for her/his kind words.

However, at the end of the day, I am left wondering how to integrate the positive data into a model that makes sense. As it stands, the authors have identified a series of potentially interesting genetic relationships that indicate a role of Dun1 beyond its known functions, but neither the relevant target nor the physiological consequences are clear from the data presented. The notion that Dun1 and Mrc1 share many, but not all phenotypes, also doesn't help to gain any mechanistic insight. As such, the manuscript unfortunately remains rather descriptive. This also becomes apparent in the abstract and the discussion, where the authors pretty much summarize their results, but do not really provide an interpretation.

We have re-written the text, focusing more on the Dun1 story, for which we have also added molecular proof of our model (Figure 7B,C). We have changed the title, Abstract and Discussion accordingly.

Some specific points:

1. I did not really understand the section about the various checkpoint factors (starting line 311). They state that according to the model, all the checkpoint factors should be essential in the hst3/4 mutant, but further down that page, they argue about suppressive effects by rad9 and/or mrc1. This needs to be better explained.

We have re-written this paragraph to make it clearer. In short, a naïve expectation would be that any mutation affecting the checkpoint would be lethal in an *Δhst3 Δhst4* background. However, this was not the case, and the results suggested a more complicated explanation, which we dissected in the next section.

2. Lines 383-386: I am not sure how the authors can conclude that Dun1 directly regulates origin firing from the notion that derepression of origin firing rescues viability.

Since in the absence of Rad53 Dun1 is no longer essential, we conclude that the lethality of *Δdun1* cells is due to an activity of Rad53. We demonstrate that the activity in question is the phosphorylation of Dbf4 and Sld3 to prevent late origin firing. Mutating the phosphorylation sites of these proteins allows late origin firing in the presence of an active Rad53 [Zegerman and Diffley, Nature 467: 474 (2010)] , and *Δdun1* viability. This demonstrates that the SS phenotype of Dun1 is due to its effect on late origin firing. We have re-written this paragraph to make it clearer. Furthermore, we have carried out qPCR experiments that directly prove that Dun1 is necessary to fire late origins normally repressed by Rad53 (Figure 8B,C).

3. There are multiple instances of sloppy expressions, genetically speaking. E.g.:
- line 241: "nor do they restore viability to Δdun1" - what is "they": the proteins or their mutants?

Corrected.

- line 275: "It was also found that they weaken the association with Pol2 subunit of Polε" - again, what is "they": the aa positions or mutants thereof?

Corrected

- line 301: "the synthetic lethality of Δdun1 and Δmrc1" - this would implicate a synthetic lethality between dun1 and mrc1, but I think the authors mean synthetic lethality between either dun1 and hst3/4 or mrc1 and hst3/4.

Corrected

Minor point:
4. Figure 3A is poorly drawn and quite confusing.

We have changed the figure.

5. "dNTPs levels" should probably be "dNTP levels" or "levels of dNTPs"

Corrected

We thank all reviewers for their insightful comments that have helped us improve our paper.
