# Supplementary material for: A novel role for Dun1 in the regulation of origin firing upon hyper-acetylation of H3K56
Source: PLoS Genet. 2021 Feb 18;17(2):e1009391. doi: 10.1371/journal.pgen.1009391 (PMC7924802; doi:10.1371/journal.pgen.1009391)
Supplement: Attachment — Submitted filename: Point by point_RR.docx [file pgen.1009391.s005.docx]

Point-by-point responses (in black) to comments by referees (in blue).

Dear Martin:

Thank you very much for submitting of revision of your Research Article entitled 'A novel role for Dun1 in the regulation of origin firing upon hyper-acetylation of H3K56' to PLOS Genetics. The reviewers were enthusiastic about the more focused version, and had only a few additional comments. Most were minor, with the exception of whether the qPCR done measure late origin replication is really a measure a origin firing.

We therefore ask you to modify the manuscript according to the review recommendations. Your revisions should address the specific points made by each reviewer.

Dear Sue,

Thanks again. Here is a response to each of the referees.

Reviewer #1: Authors responded to my comments in a satisfactory manner. The manuscript is much improved. and appropriate for PLoS Genetics. However when introducing in Discussion possible new interpretations, as suggested, authors should cite the three references I indicated in my review. Although one is already cited in anorther context, it is relevant to mention the references to support the alternative explanation and because they are relevant for this study.

We thank the reviewer for the kind words. We have now added the three references, as suggested (starting at line 363).

Reviewer #2: The authors included new qPCR data at two ARS sites to address origin firing in cells containing a Dun1 degron allele. Unfortunately, this assay can be influenced by factors other than origin firing such as passive replication. More appropriate assays would be 2D gel analyses or ChIP-seq. In the case that these assays are difficult to perform given the pandemic situation, one could at least show FACS profiles, which give a low-resolution readout of genome replication, and qPCR (DNA copy number) data across multiple early vs. late ARSs (not just two), as well as include other possible interpretation of the dun1 effects. I could not see any new data without using the Hst plasmid. please provide.

The experiments we have carried out are well-accepted in the field and have been done before in papers published in high impact journals that have undergone strict scrutiny (Crabbe et al., 2010; Poli et al., 2012; Yoshida et al., 2014). ARS305 is a bona fide early replication origin that has been used extensively as a reference in replication studies. Our control is an area known to be late replicating according to several studies. Our tested origin, ARS14-705, is located on chromosome 14 at positions 703843 to 705667. It is surrounded by two confirmed origins: ARS1427 which is a very late origin located 12,116 bp away, and ARS1428, another very late origin located 7,975 bp away. In both cases, passive replication from these origins upon treatment with HU would take far more than the 80 minutes in which we carried out our experiment (242 and 159 min, respectively at a rate of 50bp per min, as measured by Alvino et al., 2007). Given these reasoning, we do not believe that the results we have obtained could be due to passive replication (Alvino et al., 2007; Raghuraman et al., 2001; Yabuki et al., 2002).

ARS14-705 is a dubious origin that was found to be Rad53 dependent and also was found to be de-repressed in *Δhst3 Δhst4* cells (Yoshida et al., 2014).

Regarding plasmids: We have shown the lethality of strains lacking Dun1 or Mrc1 using tetrad analysis in a manner independent of the HST3-carrying plasmid. For the qPCR experiment, we constructed a degron strain of Dun1 and made use of it. These cells are unable to grow on plates containing auxin.

References:

Alvino, G.M., D. Collingwood, J.M. Murphy, J. Delrow, B.J. Brewer, and M.K. Raghuraman. 2007. Replication in hydroxyurea: it's a matter of time. *Molecular and cellular biology*. 27:6396-6406.

Crabbe, L., A. Thomas, V. Pantesco, J. De Vos, P. Pasero, and A. Lengronne. 2010. Analysis of replication profiles reveals key role of RFC-Ctf18 in yeast replication stress response. *Nature structural & molecular biology*. 17:1391-1397.

Poli, J., O. Tsaponina, L. Crabbe, A. Keszthelyi, V. Pantesco, A. Chabes, A. Lengronne, and P. Pasero. 2012. dNTP pools determine fork progression and origin usage under replication stress. *The EMBO journal*. 31:883-894.

Raghuraman, M.K., E.A. Winzeler, D. Collingwood, S. Hunt, L. Wodicka, A. Conway, D.J. Lockhart, R.W. Davis, B.J. Brewer, and W.L. Fangman. 2001. Replication dynamics of the yeast genome. *Science*. 294:115-121.

Yabuki, N., H. Terashima, and K. Kitada. 2002. Mapping of early firing origins on a replication profile of budding yeast. *Genes to cells : devoted to molecular & cellular mechanisms*. 7:781-789.

Yoshida, K., J. Bacal, D. Desmarais, I. Padioleau, O. Tsaponina, A. Chabes, V. Pantesco, E. Dubois, H. Parrinello, M. Skrzypczak, K. Ginalski, A. Lengronne, and P. Pasero. 2014. The histone deacetylases sir2 and rpd3 act on ribosomal DNA to control the replication program in budding yeast. *Molecular cell*. 54:691-697.

Reviewer #3: By focussing more on Dun1 rather than comparing Dun1 with Mrc1 and by re-writing relevant sections of the discussion, the manuscript has now become much clearer. I am now happy with the revised version. It might be worth carefully reading over the text once more for a few remaining grammatical issues.

We thank the reviewer for the kind words. We have revised the manuscript and corrected grammatical issues.
